# Cognitive performance in multiple sclerosis: what is the role of the gamma-aminobutyric acid system?

**DOI:** 10.1093/braincomms/fcad140

**Published:** 2023-05-03

**Authors:** Marijn Huiskamp, Maqsood Yaqub, Marike R van Lingen, Petra J W Pouwels, Lodewijk R J de Ruiter, Joep Killestein, Lothar A Schwarte, Sandeep S V Golla, Bart N M van Berckel, Ronald Boellaard, Jeroen J G Geurts, Hanneke E Hulst

**Affiliations:** MS Center Amsterdam, Anatomy and Neurosciences, Vrije Universiteit Amsterdam, Amsterdam Neuroscience, Amsterdam UMC location VUmc, Amsterdam, 1081 HZ, The Netherlands; Department of Radiology and nuclear medicine, Vrije Universiteit Amsterdam, Amsterdam Neuroscience, Amsterdam UMC location VUmc, Amsterdam, 1081 HZ, The Netherlands; MS Center Amsterdam, Anatomy and Neurosciences, Vrije Universiteit Amsterdam, Amsterdam Neuroscience, Amsterdam UMC location VUmc, Amsterdam, 1081 HZ, The Netherlands; Department of Radiology and nuclear medicine, Vrije Universiteit Amsterdam, Amsterdam Neuroscience, Amsterdam UMC location VUmc, Amsterdam, 1081 HZ, The Netherlands; MS Center Amsterdam, Neurology, Vrije Universiteit Amsterdam, Amsterdam Neuroscience, Amsterdam UMC location VUmc, Amsterdam, 1081 HZ, The Netherlands; MS Center Amsterdam, Neurology, Vrije Universiteit Amsterdam, Amsterdam Neuroscience, Amsterdam UMC location VUmc, Amsterdam, 1081 HZ, The Netherlands; Department of Anesthesiology, Vrije Universiteit Amsterdam, Amsterdam Neuroscience, Amsterdam UMC location VUmc, Amsterdam, 1081 HZ, The Netherlands; Department of Radiology and nuclear medicine, Vrije Universiteit Amsterdam, Amsterdam Neuroscience, Amsterdam UMC location VUmc, Amsterdam, 1081 HZ, The Netherlands; Department of Radiology and nuclear medicine, Vrije Universiteit Amsterdam, Amsterdam Neuroscience, Amsterdam UMC location VUmc, Amsterdam, 1081 HZ, The Netherlands; Department of Radiology and nuclear medicine, Vrije Universiteit Amsterdam, Amsterdam Neuroscience, Amsterdam UMC location VUmc, Amsterdam, 1081 HZ, The Netherlands; MS Center Amsterdam, Anatomy and Neurosciences, Vrije Universiteit Amsterdam, Amsterdam Neuroscience, Amsterdam UMC location VUmc, Amsterdam, 1081 HZ, The Netherlands; MS Center Amsterdam, Anatomy and Neurosciences, Vrije Universiteit Amsterdam, Amsterdam Neuroscience, Amsterdam UMC location VUmc, Amsterdam, 1081 HZ, The Netherlands; Health, Medical and Neuropsychology Unit, Institute of Psychology, Leiden University, Leiden, 2333 AK, The Netherlands

**Keywords:** multiple sclerosis, cognition, GABA-receptor, positron emission tomography, magnetic resonance spectroscopy

## Abstract

Cognitive impairment occurs in 40–65% of persons with multiple sclerosis and may be related to alterations in glutamatergic and GABAergic neurotransmission. Therefore, the aim of this study was to determine how glutamatergic and GABAergic changes relate to cognitive functioning in multiple sclerosis *in vivo*. Sixty persons with multiple sclerosis (mean age 45.5 ± 9.6 years, 48 females, 51 relapsing-remitting multiple sclerosis) and 22 age-matched healthy controls (45.6 ± 22.0 years, 17 females) underwent neuropsychological testing and MRI. Persons with multiple sclerosis were classified as cognitively impaired when scoring at least 1.5 standard deviations below normative scores on ≥30% of tests. Glutamate and GABA concentrations were determined in the right hippocampus and bilateral thalamus using magnetic resonance spectroscopy. GABA-receptor density was assessed using quantitative [^11^C]flumazenil positron emission tomography in a subset of participants. Positron emission tomography outcome measures were the influx rate constant (a measure predominantly reflecting perfusion) and volume of distribution, which is a measure of GABA-receptor density. Twenty persons with multiple sclerosis (33%) fulfilled the criteria for cognitive impairment. No differences were observed in glutamate or GABA concentrations between persons with multiple sclerosis and healthy controls, or between cognitively preserved, impaired and healthy control groups. Twenty-two persons with multiple sclerosis (12 cognitively preserved and 10 impaired) and 10 healthy controls successfully underwent [^11^C]flumazenil positron emission tomography. Persons with multiple sclerosis showed a lower influx rate constant in the thalamus, indicating lower perfusion. For the volume of distribution, persons with multiple sclerosis showed higher values than controls in deep grey matter, reflecting increased GABA-receptor density. When comparing cognitively impaired and preserved patients to controls, the preserved group showed a significantly higher volume of distribution in cortical and deep grey matter and hippocampus. Positive correlations were observed between both positron emission tomography measures and information processing speed in the multiple sclerosis group only. Whereas concentrations of glutamate and GABA did not differ between multiple sclerosis and control nor between cognitively impaired, preserved and control groups, increased GABA-receptor density was observed in preserved persons with multiple sclerosis that was not seen in cognitively impaired patients. In addition, GABA-receptor density correlated to cognition, in particular with information processing speed. This could indicate that GABA-receptor density is upregulated in the cognitively preserved phase of multiple sclerosis as a means to regulate neurotransmission and potentially preserve cognitive functioning.

## Teaser

Huiskamp *et al*. report that cognitively preserved people with multiple sclerosis showed increased GABA_A_-receptor density in the cortical and deep grey matter compared to impaired patients and controls. GABA_A_-receptor density is potentially upregulated in the cognitively preserved phase of multiple sclerosis to regulate neurotransmission and preserve cognitive functioning.

## Graphical Abstract

**Graphical abstract:**
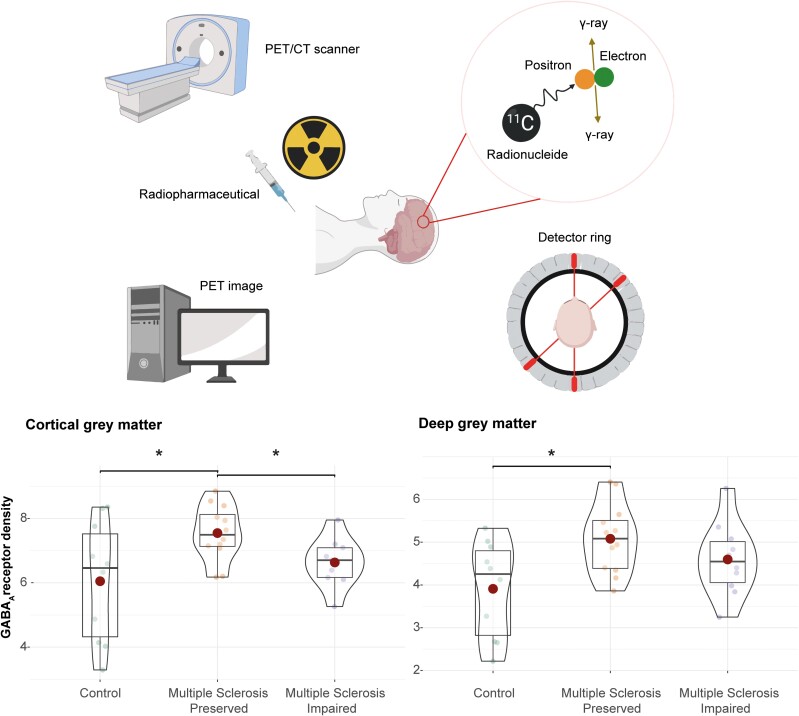

## Introduction

Cognitive impairment (CI) occurs in 40–65% of people with multiple sclerosis (PwMS) and has a severe negative impact on daily-life functioning.^1^ Previous studies using magnetic resonance imaging (MRI) have identified structural damage (i.e. lesions, white matter microstructural damage and atrophy of white and grey matter) and functional disconnection (i.e. alterations in functional activity and connectivity) as determinants of CI.^2-4^ This structural and functional disconnection may particularly affect pivotal regions such as the thalamus and hippocampus that are extensively connected to the rest of the brain.^5,6^ Despite the advances in our understanding of CI in multiple sclerosis, multimodal imaging studies so far have only been able to partially explain its occurrence,^2^ leaving its underlying neurobiological mechanisms currently still incompletely understood.

One of the potential mechanisms that can help understand CI, as well as the functional changes observed with fMRI (i.e. changes in brain activation and connectivity), are disturbances of glutamatergic and GABAergic neurotransmission.^7-9^ Glutamate and GABA are the most abundant neurotransmitters in the brain and studies have shown alterations in the synthesis, release and reuptake of these messenger molecules in multiple sclerosis.^8^ Moreover, activated microglia and macrophages in multiple sclerosis have been demonstrated to strip synapses excessively and recent studies have suggested that in multiple sclerosis GABAergic synapses are more vulnerable to phagocytosis.^10,11^ This may be highly relevant as inhibitory synapses are crucial for cognition and healthy neurotransmission.^12,13^

In order to study the glutamatergic and GABAergic systems *in vivo*, molecular imaging techniques can be employed.^14^ Concentrations of glutamate and GABA can be gauged through ^1^H-magnetic resonance spectroscopy (MRS).^15-18^ MRS studies in multiple sclerosis have shown equivocal results, with some studies reporting reduced glutamate and GABA levels in grey matter areas,^19-21^ and others showing no differences.^21-25^ In addition to measuring neurotransmitter concentrations with MRS, positron emission tomography (PET) can provide a measure of neurotransmitter receptor density by utilizing radioligands that bind to target proteins, such as GABAergic receptors.^14,26^ [^11^C]flumazenil ([^11^C]FMZ), an antagonist of the benzodiazepine site of GABA_A_-receptors, has been used to study GABA-receptor density in healthy and clinical populations since the 1980s.^26-29^ In multiple sclerosis, two studies have been performed using [^11^C]FMZ reporting contradicting results: whereas one study found indications of decreased GABA-receptor density in the cortical grey matter (GM) of persons with multiple sclerosis, the other reported increased receptor density.^30,31^ However, whether GABA-receptor density is related to cognitive impairment in multiple sclerosis and whether that coincides with changes in GABA and glutamate concentrations as measured with MRS has not been studied yet.

Therefore, the aim of this study was to determine how glutamatergic and GABAergic changes relate to cognitive functioning in multiple sclerosis *in vivo*. We employed a multimodal approach by first systematically classifying PwMS as being cognitively impaired or preserved, based on an extensive neuropsychological test battery. Second, cognitive groups were compared on thalamic and hippocampal glutamate and GABA concentrations (measured with MRS) and GABA-receptor density was compared both whole-brain and regionally using dynamic [^11^C]FMZ PET with metabolite-corrected plasma input functions.

## Methods

### Participants

Participants were recruited from the Amsterdam UMC outpatient clinic (location VUmc) and via online advertisements. PwMS between 18 and 65 years were included if they had (1) a clinically definite multiple sclerosis diagnosis according to the 2017 McDonald criteria^32^ with a relapsing-remitting or secondary-progressive disease course and (2) sufficient motor function and visual acuity to perform neuropsychological tasks. Healthy controls were matched on age, sex and education. Exclusion criteria were the presence of neurological or psychiatric illnesses (other than multiple sclerosis for PwMS), MRI contraindications and the use of corticosteroids 4 weeks before the first study visit.

Neuropsychological evaluation and MR imaging were performed during the first study visit. A subset of the participants was invited for a second visit to perform [^11^C]FMZ PET imaging if they fulfilled additional inclusion criteria: no recent use of benzodiazepines (i.e. within the last 45 days), gamma-hydroxybutyrate or other drugs that interact with the benzodiazepine receptor system. In addition, participation was precluded in the case of pregnancy, breastfeeding, insufficient haemoglobin values (i.e. < 8.0 g/dL for men and <7.0 g/dL for women) or (a history of) significant cardiac disease or exposure to previous radiation for research purposes leading to an annual cumulative dose of more than 10 millisievert.

This study protocol was approved by the institutional ethics review board and all subjects gave written informed consent before participation.

### Clinical and neuropsychological examination

Physical disability was measured using the extended disability status scale (EDSS).^33^ All subjects underwent an adjusted version of the brief repeatable test battery of neuropsychological tests,^34^ consisting of ten cognitive tests covering five cognitive domains. The Dutch version of the California verbal learning and memory test^35^ was used to assess verbal memory and learning. Outcomes were the direct free recall (i.e. total correctly remembered words of the five consecutive trials) and the long-term delayed recall (i.e. total remembered words after fifteen minutes). The location learning test^36^ was administered to measure visuospatial memory using the total displacement score of five consecutive trials as outcome. To assess working memory, the Digit span Forward and Backward and the Letter Number Sequencing test, subtests of the Wechler adult intelligence scale were used.^37^ The Word List Generation^38^ was administered to assess semantic verbal fluency. Participants were asked to name as many words belonging to the category ‘Animals’ and ‘Professions’ within 60 seconds. Finally, to assess information processing speed (IPS), card 2 of the Stroop Colour Word test^39^ and the Letter Digit Substitution Test (a variant of the symbol digit modalities test)^40^ were administered.

Cognitive test scores were corrected for age, sex and educational level when appropriate (i.e. if the variable significantly predicted the cognitive test score) using normative data of healthy controls (normative datasets vary per test with a minimum of 108 and a maximum of 173 healthy controls) and the corrected data were converted to Z-scores. CI was defined as scoring at least 1.5 SD below norm data on ≥30% of tests, but only when at least two out of five cognitive domains were affected (i.e. verbal memory, visuospatial memory, working memory, verbal fluency or IPS).^41,42^ Otherwise, PwMS were considered cognitively preserved (CP). Domain-specific Z-scores were obtained by averaging Z-scores of all individual tests belonging to each specific cognitive domain.

### MR imaging and analysis

MR imaging was performed on a whole body 3 T MRI-scanner (GE Discovery, Milwaukee, WI, USA) using a 32-channel head coil. The protocol included a three-dimensional (3D) T1-weighted fast spoiled gradient echo scan (FSPGR; repetition time (TR) = 8.22 ms, echo time (TE) = 3.22 ms, inversion time (TI) = 450 ms, flip angle 12°, 1.0 mm sagittal slices with 0.94 × 0.94 mm^2^ in-plane resolution) and a 3D fluid-attenuated inversion recovery (FLAIR; TR = 8000 ms; TE = 128 ms; TI = 2343 ms; 1.2 mm sagittal slices; 0.98 × 0.98 mm^2^ in-plane resolution).

White matter lesions were automatically segmented on the FLAIR image and filled on the 3D-T1 images according to previously published methods.^43,44^ FSL’s Sienax (fsl.fmrib.ox.ac.uk) was used to obtain volumes of white and grey matter (WMV, GMV) and FIRST was used to obtain total deep grey matter volume, thalamic volume and right and total hippocampal volume. All volumes were normalized for head size.

### MR spectroscopy and analysis

All participants underwent MRS. The volume-of-interest (VOI) for the right hippocampus was 10 ml [superior—inferior (SI) = 14 mm, right—left (RL) = 20 mm, anterior—posterior (AP) = 36 mm] and for the bilateral thalamus 10.8 ml (SI = 16 mm, RL = 28 mm, AP = 24 mm). Both the hippocampal and thalamic VOIs were manually positioned using the 3D T1-weighted images and oblique-transversal reconstructions (see example in Supplementary Fig. 1). High-order shimming was performed for both VOIs to optimize local field homogeneity. Single voxel point resolved spectroscopy (PRESS, TR/TE =3000/35 ms, 64 averages) and Mescher–Garwood point resolved spectroscopy sequences (MEGA-PRESS, TR/TE = 1800/68 ms, 128 averages) were acquired from both the right hippocampal and bilateral thalamus VOIs.

PRESS data were analysed with LCModel version 6.3–1^45^ using water scaling, a standard basis set consisting of 17 metabolites including simulated macromolecules and lipids. For this study, we focused on glutamate (Glu), total *N*-acetylaspartate (NAA, including contributions from *N*-acetyl-aspartyl-glutamic acid) and total creatine signals (Cr, including contributions from phosphocreatine). The spectral fitting range was from 4.0 ppm to 0.2 ppm (Supplementary Fig. 1). All spectra were visually inspected and excluded if the full-width half maximum (FWHM) was >0.1 ppm (i.e. 12 Hz) or the signal-to-noise ratio (SNR) was <5 in combination with a visually confirmed low-quality spectrum.

The MEGA-PRESS data were analyzed with GANNET (version 3.0),^46^ which estimates GABA concentrations relative to water signal. Because macromolecules contribute to the GABA-signal, it will be referred to as GABA^+^ and concentrations are expressed as institutional units (i.u.). All GANNET output was visually inspected and spectra were excluded in case of visually poor GABA^+^ fits and a GABA^+^ fit error exceeding 20.

The fraction of WM, GM and cerebrospinal fluid (CSF) in the VOIs was calculated by segmentation of the lesion-filled 3D T1-weighted image using Sienax and FIRST. These fractions were used to correct the water-scaled concentrations for the presence of CSF and tissue fractions in the VOI.^45^

### PET imaging and analysis

A subset of participants received dynamic [^11^C]FMZ PET on a Philips Ingenuity TF-128 PET/computed tomography (CT) scanner (Philips Healthcare, Best, The Netherlands). Before the PET scan, a low-dose CT scan was performed for attenuation correction purposes. An intravenous bolus injection of 370 ± 37 Mega Becquerel [^11^C]FMZ was administered at the start of the dynamic PET scan of 60 minutes. PET images were reconstructed using Blob-OS-TF reconstruction,^47^ as provided by the vendor, and consisted of 19 frames (1 × 15 s, 3 × 5 s, 3 × 10 s, 4 × 60 s, 2 × 150 s, 2 × 300 s, 4 × 600 s). An arterial line, inserted in a radial artery before scanning, was used to measure whole blood activity concentration continuously and discrete blood samples were also obtained at predetermined time points (i.e. at 5, 10, 15, 20, 30, 40 and 60 minutes). These discrete blood samples were used to calibrate the (online) blood sampler counts and also to correct the blood sampler for plasma/whole-blood ratios, metabolite fractions and delay to obtain a metabolite-corrected plasma input curve. Fractional concentrations of hydrophilic metabolites and unchanged (lipophilic) [^11^C]FMZ were determined by solid-phase extraction of plasma followed by high-performance liquid chromatography.^48,49^

In order to account for partial volume effects (PVE), an established iterative deconvolution method in combination with denoising was used, which has been previously validated in [^11^C]FMZ studies.^50^ Then, the lesion-filled 3D T1-weighted MRI images were coregistered to the summed [^11^C]FMZ images using Vinci v4.66.^51^ Coregistered images were segmented using PVE-lab^52^ and Alexander-Hammers template, consisting of 67 VOIs.^53^ These VOIs were projected onto the dynamic PET images to obtain regional time activity curves (TACs).

From previous pharmacokinetic studies, it is known that both one-tissue and two-tissue plasma input models can be used to accurately analyse [^11^C]FMZ data, but that one-tissue models are usually preferred due to fewer outliers while maintaining high accuracy.^48^ As we also observed fewer outliers (i.e. coefficient of variation of more than 25%) in the regional TACs using the single-tissue model, this model was the optimal choice for the analysis (for a formal comparison of one-tissue and two-tissue models, see Supplementary Data 1). Outcome measures were influx rate constant (*K_1_*), a measure that predominantly reflects perfusion (i.e. assuming comparable extraction fraction across the brain and subjects) and volume of distribution (*V_T_*), which represents the ratio between tracer in the tissue and in the blood and is an estimate of GABA-receptor density. *K_1_* and *V_T_* values were extracted for cortical GM, deep GM, the thalamus and hippocampus.

### Statistical analysis

All statistical analyses were performed using IBM SPSS 26 (Armonk, NY: IBM Corp) and RStudio 2021 (Boston, MA, USA). Comparisons of demographic, clinical, volumetric, MRS and PET variables were done using Welch’s t-tests in case of multiple sclerosis versus healthy control (HC) comparisons, Welch’s ANOVAs when comparing CP, impaired and HC groups or *χ*^2^ tests for dichotomous variables. Correlational analyses between cognition and imaging (i.e. MRS and PET) measures were performed using Pearson’s R or Spearman’s *ρ* in case of non-normality. Results were significant at *P* < 0.05 and correlations were reported both with and without Holm’s step-down correction for multiple comparisons. Variables were checked for normality using Kolmogorov–Smirnov tests and histogram inspection.

As a *post hoc* analysis, the correlations between *K_1_* and *V_T_* and cognitive scores were repeated in the cognitively impaired and preserved groups separately, for which Spearman’s *ρ* was used.

## Results

### Demographic, clinical and volumetric data

A total of 60 PwMS and 22 healthy controls participated in the current study. Of the PwMS, 20 showed CI while 40 were CP. Age, sex and educational level were equally distributed between multiple sclerosis and HC groups (Table 1). PwMS had significantly lower volumes for total GM, deep GM, hippocampus and thalamus compared to healthy controls, with cognitively impaired PwMS having the lowest volumes (Table 2).

**Table 1:** Demographic and clinical data across groups. Significant values are shown in **bold**.

Total sample	HC (*n* = 22)	CP (*n* = 40)	CI (*n* = 20)	*P*-value
Age (years)	45.6 (11.0)	45.0 (9.5)	46.6 (9.8)	0.84
Sex (F/M)	17/5	32/8	16/4	0.96
Educational level (5/6/7)	7/11/4	10/21/9	4/13/3	0.83
RRMS/SPMS/unknown	–	35/4/1	16/4/0	0.45
Disease duration (years)	–	8.7 (6.2)	12.1 (7.8)	0.10
Medication (first line, second line, none), %	–	30/27.5/42.5	15/55/35	0.11
Medication type (IF-β, GA, DMF, TF, NTZ, FI, OCR, none), %	–	5/10/10/5/17.5/7.5//2.5/42.5	5/5/0/5/15/10/25/35	–
EDSS	–	4.0 [3.5–5.0]	4.25 [3.1–5.5]	0.52

PET sample	HC (*n* = 10)	CP (*n* = 12)	CI (*n* = 10)	*P*-value
Age (years)	45.2 (13.5)	42.3 (10.6)	45.5 (10.2)	0.76
Sex (F/M)	7/3	9/3	8/2	0.88
Educational level (5/6/7)	2/5/3	3/8/1	3/6/1	0.67
RRMS/SPMS/unknown	–	12/0	9/1	–
Disease duration (years)	–	7.4 (6.9)	12.8 (9.3)	0.15
Medication (first line, second line, none), %	–	41.7/25/33.3	10/60/30	0.09
Medication type (IF-β, GA, DMF, TF, NTZ, FI, OCR, none), %	–	0/25/8.3/8.3/16.7/8.3/0/33.4	10/0/0/0/10/10/40/30	–
EDSS	–	4.25 [3.5–5.9]	4.5 [3.0–5.5]	0.63

Data are presented as mean (SD) or median [IQR] in case of non-normality. HC = healthy control; CP = cognitively preserved multiple sclerosis; CI = cognitively impaired multiple sclerosis; RRMS = relapsing-remitting MS; SPMS = secondary progressive MS; IF-β = interferon-gamma; GA = glatiramer acetate; DMF = dimethyl fumarate; TF = teriflunomide; NTZ = natalizumab; FI = fingolimod; OCR = ocrelizumab; EDSS = expanded disability status scale.

**Table 2:** Volumetric data across groups. Significant values are shown in **bold**.

Volumes total sample (ml)	HC (*n* = 22)	CP (*n* = 40)	CI (*n* = 20)	*P*-value
NWMV	716.2 (39.7)	706.0 (38.7)	684.1 (43.4)	**0**.**031**
NLV	–	12. 1 [7.7–16.5]	25.1 [15.4–46.4]	**0**.**001**
NGMV	835.6 (62.0)	809.4 (63.5)	743.7 (76.3)	**<0**.**001**
NDGMV	63.5 (5.7)	60.5 (5.2)	52.3 (7.0)	**<0**.**001**
Right hippocampus	5.1 (0.6)	4.9 (0.7)	4.3 (1.0)	**0**.**009**
Bilateral hippocampus	10.0 (1.2)	9.5 (1.3)	8.5 (1.3)	**<0**.**001**
Bilateral thalamus	21.5 (2.0)	20.1 (2.0)	16.9 (2.5)	**<0**.**001**

Volumes PET sample (ml)	HC (*n* = 10)	CP (*n* = 12)	CI (*n* = 10)	*P*-value
NWMV	698.9 (25.4)	698.5 (39.5)	667.9 (46.9)	0.201
NLV	–	15.8 [11.4–26.1]	25.1 [11.1–47.9]	0.190
NGMV	846.2 (59.9)	790.0 (73.2)	755.0 (87.0)	**0**.**033**
NDGMV	64.6 (3.7)	57.6 (6.0)	51.5 (7.6)	**<0**.**001**
Right hippocampus	5.3 (0.5)	4.7 (0.6)	4.2 (0.9)	**0**.**004**
Bilateral hippocampus	10.4 (1.1)	8.9 (1.3)	8.3 (1.3)	**0**.**002**
Bilateral thalamus	21.5 (1.2)	19.3 (2.1)	16.6 (2.8)	**<0**.**001**

HC = healthy control; CP = cognitively preserved multiple sclerosis; CI = cognitively impaired multiple sclerosis; NWMV = normalized white matter volume; NLV = normalized lesion volume; NGMV = normalized grey matter volume; NDGMV = normalized deep grey matter volume. Pairwise comparisons in total sample: ^a^HC versus CI, ^b^CP versus CI and ^c^HC versus CP. NWMV: 0.007^a^; NGMV: < 0.001^a^, < 0.001^b^; NDGMV: < 0.001^a^, < 0.001^b^; right hippocampus: < 0.001^a^, 0.006^b^, 0.044^c^; Bilateral hippocampus: < 0.001^a^, < 0.001^b^, 0.014^c^; thalamus: < 0.001^a^, < 0.001^b^. Pairwise comparisons in PET sample: ^d^HC versus CI, ^e^CP versus CI and ^f^HC versus CP. NGMV: 0.010^d^; NDGMV: < 0.001^d^, 0.023^e^, 0.011^f^; right hippocampus: 0.001^d^, 0.048^f^; bilateral hippocampus: < 0.001^d^, 0.005^f^; Thalamus: < 0.001^d^, 0.006^e^, 0.023^f^.

### MRS results

According to the quality criteria stated above, several spectra were excluded (for an overview of the numbers of excluded spectra per group and metabolite, see Supplementary Table 1). After excluding these poor quality spectra, the FWHM (i.e. PRESS quality measure) and GANNET fit error (i.e. MEGA-PRESS quality measure) were equal between groups. The SNR was lower in cognitively impaired PwMS than in the other groups, despite being well above the cut-off value of 5 (Supplementary Table 2). No differences in any of the metabolite concentrations (i.e. Cr, NAA, Glu or GABA^+^) were observed between the groups (i.e. multiple sclerosis and HC and cognitively impaired and preserved) in either the hippocampus or thalamus (Supplementary Table 2).

### PET results

**Figure 1:**
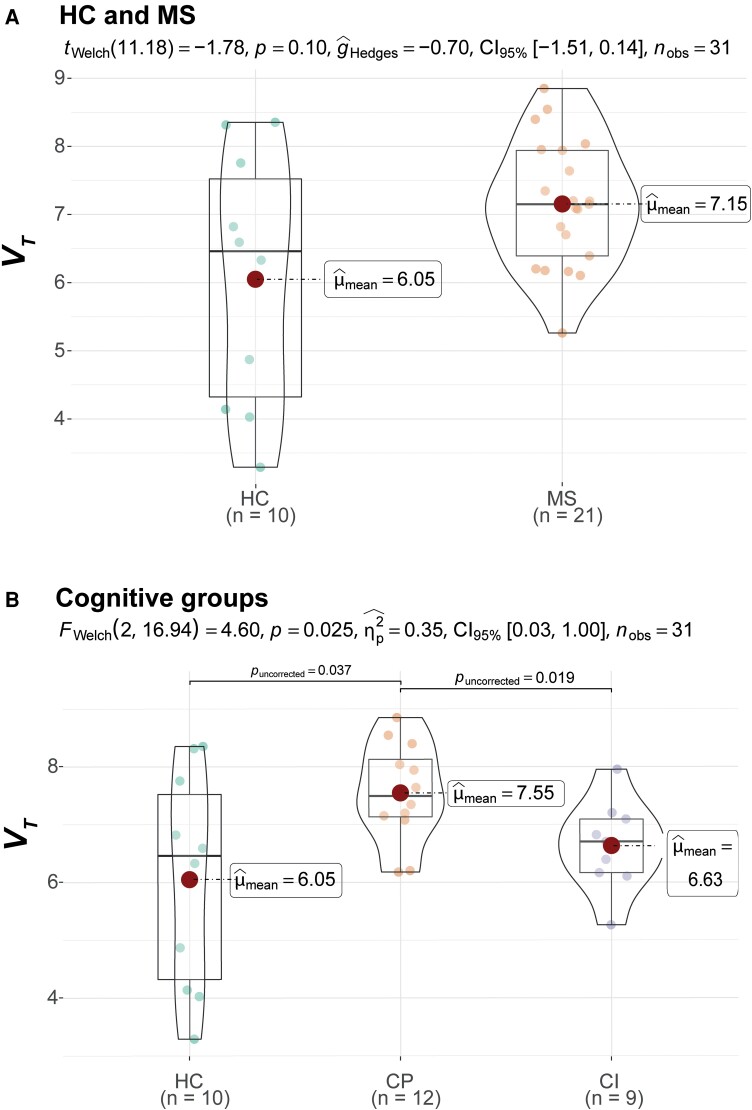
**Cortical GM volume of distribution (*V_T_*) differences between HC and multiple sclerosis groups**. Figures are violin plots with boxplots and distribution of data. Statistical comparisons of multiple sclerosis versus HC are shown in **A** using Welch’s *t*-test (*t_welch_*) while comparisons between HC, CP and CI PwMS are shown in **B** using Welch’s ANOVA (*F_welch_*). HC = healthy control; MS = multiple sclerosis; CP = cognitively preserved multiple sclerosis; CI = cognitively impaired multiple sclerosis.

**Figure 2:**
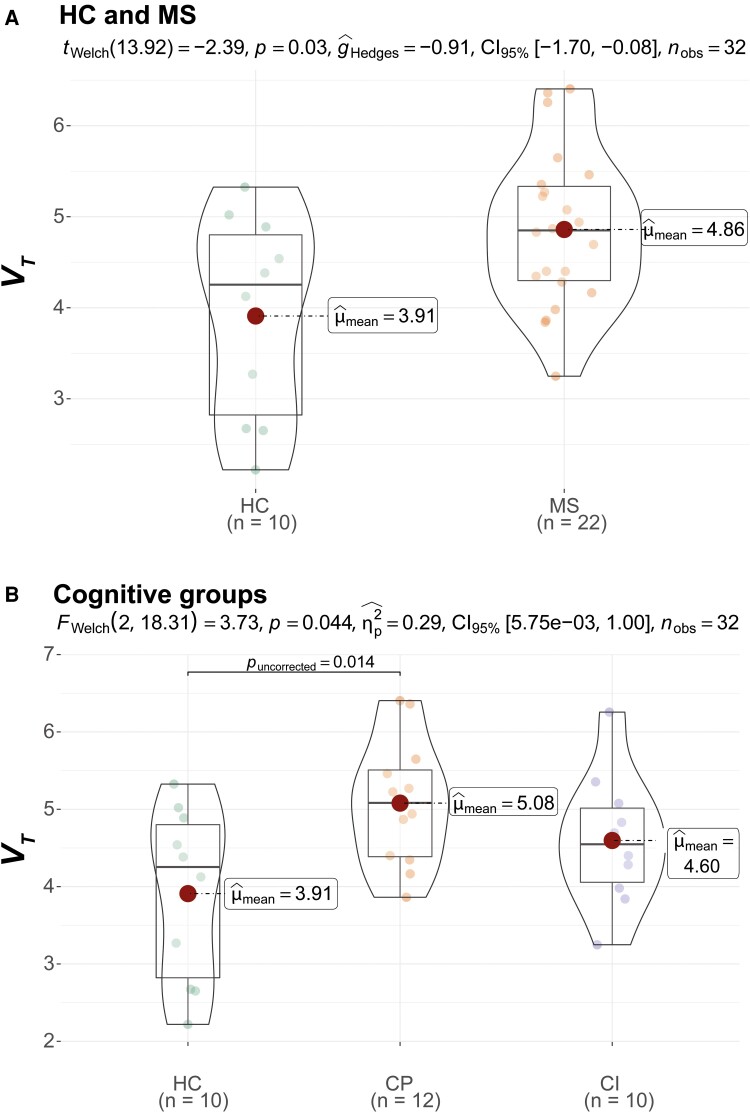
**Deep GM volume of distribution (*V_T_*) differences between HC and multiple sclerosis groups**. Figures are violin plots with boxplots and distribution of data. Statistical comparisons of multiple sclerosis versus HC are shown in **A** using Welch’s *t*-test (*t_welch_*) while comparisons between HC, CP and CI PwMS are shown in **B** using Welch’s ANOVA (*F_welch_*). HC = healthy control; MS = multiple sclerosis; CP = cognitively preserved multiple sclerosis; CI = cognitively impaired multiple sclerosis.

**Figure 3:**
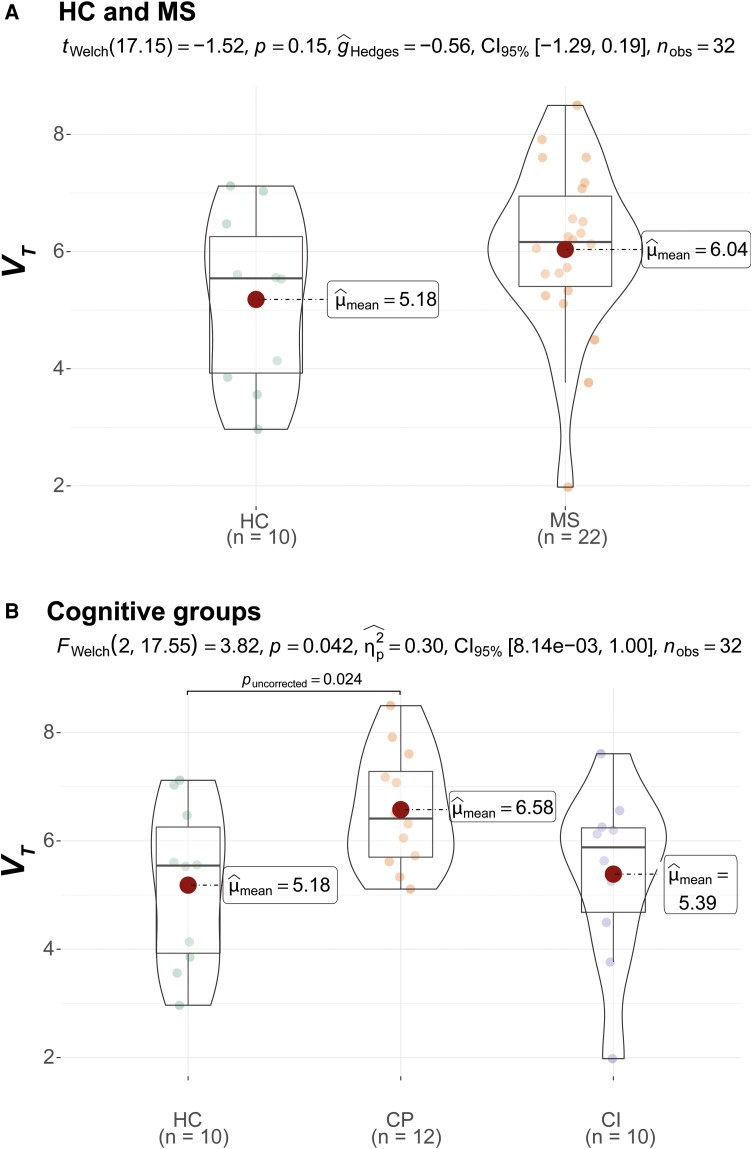
**Hippocampal volume of distribution (*V_T_*) differences between HC and multiple sclerosis groups**. Figures are violin plots with boxplots and distribution of data. Statistical comparisons of multiple sclerosis versus HC are shown in **A** using Welch’s *t*-test (*t_welch_*) while comparisons between HC, CP and CI PwMS are shown in **B** using Welch’s ANOVA (*F_welch_*). HC = healthy control; MS = multiple sclerosis; CP = cognitively preserved multiple sclerosis; CI = cognitively impaired multiple sclerosis.

In total, 31 PwMS and 13 healthy controls underwent [^11^C]FMZ PET scanning. Due to data acquisition and analysis issues (i.e. unreliable input function, *N* = 9; poor atlas registration, *N* = 2; and scanned under wrong protocol, *N* = 1), nine PwMS and three healthy controls were excluded from the analysis. The total sample thus consisted of 22 PwMS (12 CP and 10 impaired) and 10 healthy controls, who did not differ on demographic parameters (Table 1). PVE-corrected results are shown here (for uncorrected data, see Supplementary Figs. 4 and 5). PwMS, compared to HCs, showed higher *V_T_* values in deep GM (*P* = 0.030, Fig. 2A) and lower K1 values in the thalamus (*P* = 0.020, Supplementary Fig. 2). When comparing impaired and preserved PwMS to healthy controls, the CP group showed significantly higher *V_T_* than healthy controls on cortical GM (*P* = 0.037, Fig. 1B), deep GM (*P* = 0.014, Fig. 2B) and hippocampus (*P* = 0.024, Fig. 3B). In the comparison between CP and impaired PwMS, preserved PwMS also showed higher *V_T_* than the impaired group in cortical GM (*P* = 0.019, Fig. 1B). For all PVE-corrected group comparisons on *K_1_* and *V_T_*, please see Supplementary Figs 2 and 3, respectively. Group comparisons on *K_1_* and *V_T_* without PVE-correction are displayed in Supplementary Figs 4 and 5, respectively.

### Correlations with clinical and cognitive data: MRS and PET

**Figure 4:**
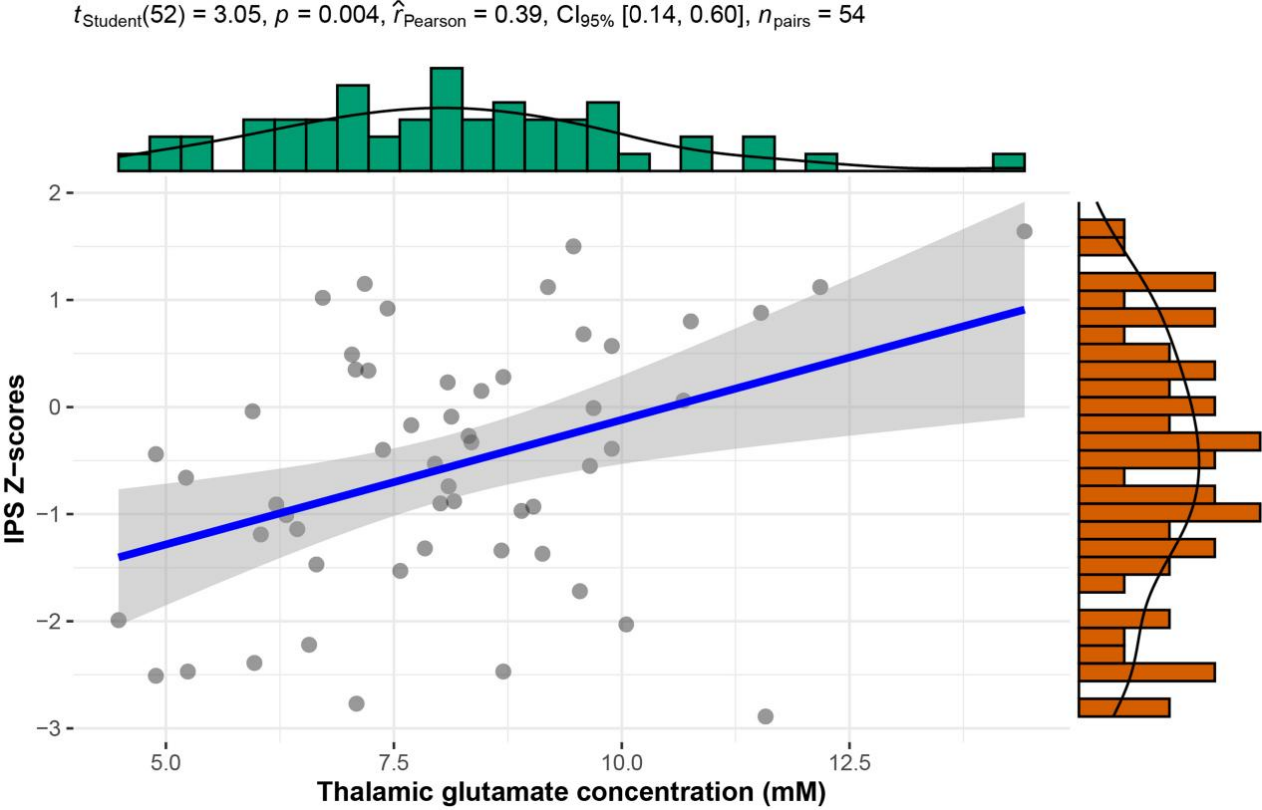
**Relationship between thalamic glutamate and IPS in multiple sclerosis.** Pearson’s correlation with scatterplot and fit line including 95% confidence interval (grey area).

In the MRS analysis, the HC hippocampal GABA^+^ concentration related to verbal memory (*r* = 0.63, *P* = 0.006). In multiple sclerosis, significant correlations were found between hippocampal GABA^+^ concentrations and visuospatial memory (*r* = 0.36, *P* = 0.017) and between thalamic glutamate and IPS (*r* = 0.39, *P* = 0.004, Fig. 4). However, none of these correlations in HC or PwMS remained significant after multiple comparison correction.

**Figure 5:**
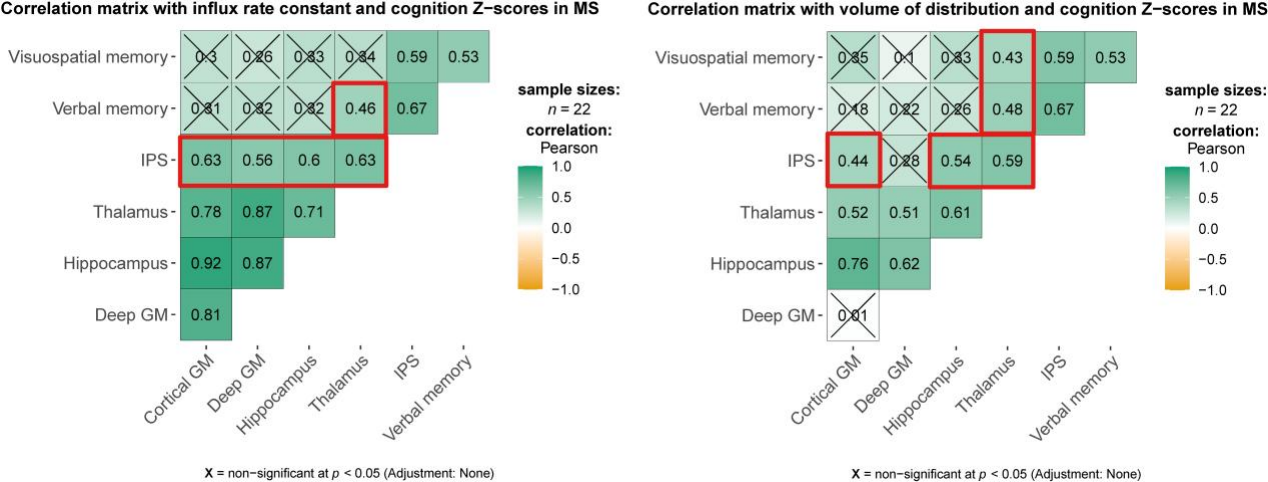
**Pearson’s correlation matrices showing positive correlations of cognition z-scores with influx rate constant and volume of distribution.** Correlation coefficients of PET measures and cognition are indicated with a bold border. MS = multiple sclerosis; IPS = information processing speed; GM = grey matter.

In the PET analysis, none of the measures correlated to EDSS scores. However, positive correlations were observed between all PET measures (except those of the deep GM) and IPS in the multiple sclerosis group only (Fig. 5). Moreover, thalamus *K_1_* correlated with verbal memory (*r* = 0.46, *P* = 0.034) and thalamus *V_T_* correlated with verbal (*r* = 0.48, *P* = 0.023) and visuospatial memory (*r* = 0.43, *P* = 0.046). These relationships did not occur for other cognitive domains and no significant correlations were observed in the HC group. After multiple comparison correction, the correlation between the *K_1_* of the cortical GM and IPS remained significant.

*Post hoc*, we investigated whether the significant correlation patterns between PET measures and cognition, as seen in the multiple sclerosis group, existed in the cognitively impaired and preserved groups separately. This showed that the relationships were not present in the CP group. However, in the impaired group, the relationship between IPS and *K_1_* of total GM (*ρ* = 0.65, *P* = 0.042) and *K_1_* of cortical GM (*ρ* = 0.69, *P* = 0.026) was significant and several other PET measures showed borderline significant correlations with IPS (cortical GM *V_T_*: *ρ* = 0.61, *P* = 0.060); hippocampus *K_1_*: *ρ* = 0.63, *P* = 0.063; hippocampus *V_T_*: *ρ* = 0.61, *P* = 0.062; thalamus *V_T_*: *ρ* = 0.60, *P* = 0.069).

## Discussion

This study set out to determine how measures of the glutamatergic and GABAergic systems play a role in cognitive functioning in PwMS. We found no differences in the MRS-measured metabolite levels of glutamate and GABA^+^ in either the hippocampus or thalamus between CP and impaired PwMS and healthy controls. However, using [^11^C]FMZ PET imaging, increased GABA-receptor density was found in the cortical and deep GM and the hippocampus of CP PwMS. In addition, glutamate concentration in the thalamus as measured with MRS and global measures of perfusion and GABA-receptor density showed positive correlations with cognitive functioning, particularly with IPS in PwMS, but not in healthy controls. These results suggest that changes in the glutamatergic and GABAergic systems are relevant for cognitive functioning in multiple sclerosis, and, more specifically, that increased GABA-receptor density in the CP phase of the disease may be a transient process that can only be maintained for a limited time period.

The increased GABA-receptor density was primarily observed in the CP group compared to healthy controls. The volume of distribution in the cognitively impaired group, although showing somewhat elevated levels, was not significantly higher than in healthy controls. Despite the cross-sectional design of our work, this suggests an initial increase in GABA-receptor density in the CP phase of multiple sclerosis that is only temporarily present and disappears as patients progress to the impaired phenotype. This inverted-U pattern of upregulated neural resources in CP multiple sclerosis that disappears when impairment manifests, has been described extensively in the field of functional activity and connectivity in multiple sclerosis. For example, multiple studies have shown increased functional activation of task-related networks in CP PwMS as compared to impaired PwMS or controls, whereas in impaired PwMS activation levels decline, sometimes even below HC levels.^54-56^ This has generally been interpreted as a form of functional reorganization, either being adaptive or maladaptive.^57^ Studies on functional connectivity (i.e. co-fluctuations in activation between brain regions or networks) have shown the same pattern of initial in- and subsequent decrease of connectivity in multiple sclerosis,^58,59^ although this can vary across studies.^59^ These varying study results have led to the postulation of the ‘network collapse’ hypothesis, which holds that the combination of structural and functional changes eventually lead to cognitive and network deterioration in multiple sclerosis.^59^ Considering the crucial role of GABAergic synapses and receptors in network, clinical and cognitive functioning,^12,13,60^ it is possible that the GABA-receptor density changes observed in this study form a link in the cascade of events leading to this network collapse.

Whether the increased GABA-receptor density that was observed in this study leads to increased inhibitory neurotransmission and how that relates to patterns of functional activation and connectivity needs to be determined. Moreover, it will be imperative to obtain a similar measure of the glutamatergic system, using a PET tracer for glutamate receptors, as it is possible that both inhibitory and excitatory neurotransmission are upregulated simultaneously in CP PwMS, preserving a balance between excitation and inhibition.^8^ Unfortunately, tracers for glutamate receptors are currently unavailable or suffer from methodological issues.^61^ A previous [^11^C]FMZ PET study in multiple sclerosis observed a decreased total number of GABA-receptors in multiple sclerosis.^30^ Although this seems to conflict with our results, the adopted methodologies differed as the aforementioned study used a partial saturation protocol whereas we used arterial sampling. Moreover, the studied samples also showed differences as the study by Freeman and colleagues^30^ contained more patients with longer disease duration, higher disability and secondary-progressive disease course. Despite these differences, Freeman *et al.*^30^ also observed positive correlations between the number of GABA-receptors and cognitive functioning. This is in line with our observations and corroborates the idea that increased GABA-receptor density contributes to preserved cognition in multiple sclerosis.

Alternatively, the increased GABA-receptor density in the CP group might be a response to neuroinflammation. Inflammation and the GABAergic system have been described to interact extensively in both the healthy and multiple sclerosis brain.^8,62^ For instance, in the presence of inflammatory cytokines, GABA_A_-receptors are upregulated *in vitro*^63^ and this was corroborated in a recent study *in vivo* where increased GABA-receptor density was observed in multiple sclerosis which correlated with innate immune activity.^31^ For our current data this has two implications. First, definite conclusions about whether our CP PwMS experienced more inflammation than impaired PwMS cannot be made as no direct measure of inflammation was included in this study (e.g. with the use of gadolinium-enhanced MRI). However, PwMS were precluded from participation if they reported a relapse or corticosteroid use within 4 weeks of study onset, consequently excluding PwMS with clinically acute inflammation. In addition, the majority of both groups used immunomodulatory treatment (Table 1). Second, inflammation is known to negatively affect cognition.^64^ If increased GABA-receptor density in our CP sample resulted from higher levels of inflammation, a negative instead of a positive relationship with cognition would be expected. Therefore, the effect of neuroinflammation alone, albeit important to consider, seems insufficient to fully explain the current results.

The MRS analysis did not show altered levels of GABA^+^ or glutamate between groups. This is not uncommon as previous studies in multiple sclerosis have reported lower,^19,20^ unchanged^25^ or sometimes even higher^24^ levels of glutamate and GABA levels in GM. As we have optimized our methodology for the quantification of glutamate and GABA^+^ (e.g. by performing water scaling, excluding low-quality spectra, using a spectral editing sequence for GABA and performing PVE-correction), we can only conclude that the levels of glutamate and GABA^+^ in this multiple sclerosis sample are unaltered. As the GABA^+^ concentration in the hippocampus and thalamus did not correlate with the PET measures in these regions (data not shown), this may be due to the fact that GABA MRS and [^11^C]FMZ PET quantify different elements of the GABAergic system. This could also mean that the GABA-receptor system is more sensitive to multiple sclerosis-induced alterations than neurotransmitter concentrations, but that requires further examination. Finally, because GABA-receptor measures are associated with cognitive functioning, but not with a clinical measure such as the EDSS, [^11^C]FMZ PET imaging may be able to provide unique explanatory information by quantifying physiological processes involved in cognitive functioning.^26^

Considering the *K_1_* results, we noted lower perfusion in the thalamus of PwMS compared to controls. This is consistent with previous literature reporting reduced cerebral perfusion in multiple sclerosis, both in the deep GM,^65^ thalamus^66^ and in the cortex.^67^ Then, we found a positive correlation between cortical *K_1_* and IPS, that survived multiple comparison correction. Previous studies in multiple sclerosis have reported correlations between cerebral perfusion and cognitive performance as well,^65^ and also with IPS in particular.^67^ It is thus possible that reduced perfusion, reflected by a lower *K_1_*, contributed to lower IPS scores in the multiple sclerosis group. Although perfusion dynamics may thus contribute to cognitive performance and influence GABA-receptor binding, it is most likely a phenomenon non-specific to GABA-receptors.

Some limitations apply to this work. First, although the study aimed to determine the involvement of the glutamatergic and GABAergic systems in relation to cognition in multiple sclerosis *in vivo*, the complex cycle of glutamatergic and GABAergic neurotransmission could only be described in part. Nevertheless, within the realm of *in vivo* measurements of these systems, we have combined the current state-of-the-art measures. Future studies should consider including MRS imaging which allows the quantification of metabolites in a much larger area than in a single voxel.^68^ Second, this study has a cross-sectional design and therefore any inferences about initial increases in GABA-receptor density that progress in a reduction will need longitudinal validation. Third, we fully acknowledge how important it is to account for the effects of MS-related structural damage in this study (e.g. white matter lesions or GM volume loss). To minimize these factors we have applied partial volume correction in both the MRS and PET analysis. However, even after partial volume correction, we cannot completely exclude the possibility that structural damage may have had an effect on our results. Confounding effects of age and sex have been minimized as well by group matching and by correcting the neuropsychological test scores for these effects. But, due to sample size limitations, these demographic factors have not been included in the statistical analyses as covariates. Future studies with larger samples can hopefully disentangle the cognitive, demographic and clinical variability that was inherently present in our sample on a more fine-grained level. This would also allow a more detailed regional analysis of GABA-receptor binding, which could help identify the regions with the largest changes in receptor density. As this was the first exploration of both the glutamatergic and GABAergic role in CI in multiple sclerosis, this was out of the scope of this study. Finally, future work should relate the current findings to functional activation during a cognitive task or to functional connectivity during a resting-state to determine how GABA-ergic changes underlie network changes.

In conclusion, this study is unique in its comprehensive characterization of cognitive functioning in PwMS in combination with an innovative, combined approach of MRS and PET investigations to study the glutamatergic and GABAergic system. Increased GABA-receptor density was observed in CP PwMS that was not seen in cognitively impaired PwMS. In addition, GABA-receptor density correlated to cognition, in particular with IPS. These findings suggest that GABA-receptor density is transiently upregulated in CP PwMS and can only be sustained temporarily or only in the presence of a limited amount of pathology. Once the amount of pathology exceeds that limit, it is possible that GABA-receptor density returns to its prior level. If future studies corroborate the beneficial effects of increased GABA-receptor density on cognition, this may pave the way for pharmacological interventions aimed at stimulating GABA-receptor function and preserving cognition in multiple sclerosis.

## Supplementary Material

fcad140_Supplementary_Data

## Data Availability

The data analysed in the current study are available from the corresponding author on reasonable request.

## Abbreviations

[^11^C]FMZ =: [^11^C]-flumazenil
AP =: anterior–posterior
CI =: cognitive impairment
CP =: cognitively preserved
Cr =: creatine
CSF =: cerebrospinal fluid
CT =: computed tomography
EDSS =: expanded disability status scale
FLAIR =: fluid-attenuated inversion recovery
FSPGR =: fast spoiled gradient echo scan
FWHM =: full-width half maximum
Glu =: glutamate
GM =: grey matter
Hz =: hertz
i.u. =: institutional units
*K_1_* =: influx rate constant
MEGA-PRESS =: Mescher–Garwood point resolved spectroscopy
MRI =: magnetic resonance imaging
MRS =: magnetic resonance spectroscopy
NAA =: total *N*-acetylaspartate
PET =: positron emission tomography
PPM =: parts per million
PRESS =: point resolved spectroscopy
PVE =: partial volume effects
PwMS =: persons with multiple sclerosis
RL =: right-left
RRMS =: relapsing-remitting multiple sclerosis
SI =: superior–inferior
SNR =: signal-to-noise ratio
SPMS =: secondary progressive multiple sclerosis
TE =: echo time
TR =: repetition time
VOI =: volume of interest
*V_T_* =: volume of distribution
WM =: white matter
