# Supplementary material for: Cognitive performance in multiple sclerosis: what is the role of the gamma-aminobutyric acid system?
Source: Brain Commun. 2023 May 3;5(3):fcad140. doi: 10.1093/braincomms/fcad140 (PMC10174207; doi:10.1093/braincomms/fcad140)
Supplement: fcad140_Supplementary_Data [file fcad140_supplementary_data.pdf]

Supplementary data

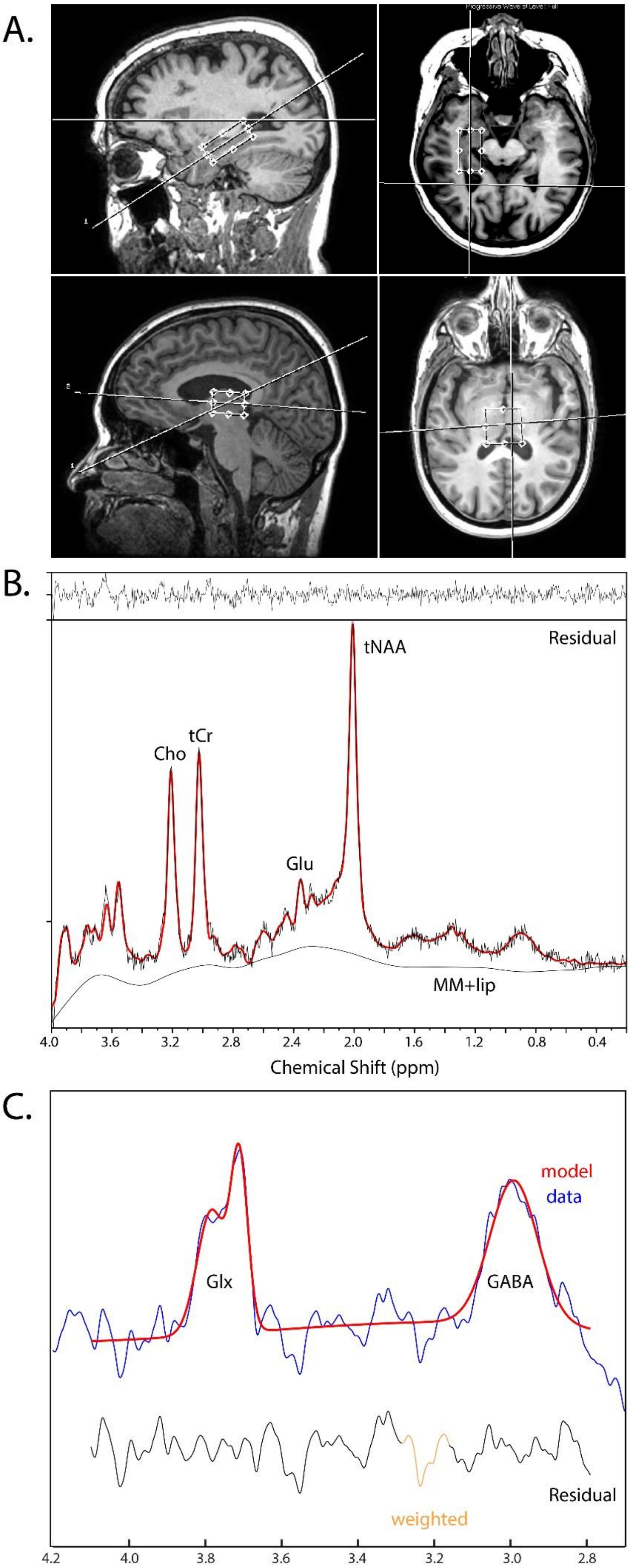

A.
B.
Residual
tNAA
Cho
tCr
Glu
MM+lip
4.0
3.6
3.2
2.8
2.4
2.0
1.6
1.2
0.8
0.4
Chemical Shift (ppm)
C.
model
data
Glx
GABA
Residual
weighted
4.2
4.0
3.8
3.6
3.4
3.2
3.0
2.8

Supplementary figure 1 | Glutamate and GABA MRS acquisition and analysis. (A) MRS VOI placement in right hippocampus and bilateral thalamus. **(B)** Representative PRESS spectrum from an MS patient and **(C)** Representative MEGA-PRESS fits of GABA and glutamine + glutamate (Glx) signal from an MS patient.

Supplementary data 1 | Comparison of single- and two-tissue pharmacokinetic models for [^{11}C]FMZ analysis

In order to compare the goodness-of-fit of the single-tissue (1T2k_Vb) and two-tissue (2T4k_Vb) compartmental models with an additional parameter correction for blood volume fraction, the Akaike Information Criterion (AIC) was used.[1] For each TAC of each participant (N=990), the AIC values of both models were compared and the model with the lower value was considered optimal. This showed that in 498/990 TACs (50.3%) the 1T2k_Vb model was preferred and in 492 TACS (49.7%) the 2T4k_Vb was better. Then, we compared the number of outliers for the V_T of each model (i.e. coefficient of variation of >25%). In the 1T2k_Vb model no outliers occurred, while in the 2T4k_Vb model 108 (10.9%) V_T values were outliers. Finally, the V_T values of both models showed a very high Pearson's correlation (r = 0.98), corroborating their similar performance. Due to the nearly equal performance of both models and the higher number of outliers in the 2T4k_Vb model, the simpler 1T2k_Vb model was chosen for the current analysis.

Supplementary table 1 | Excluded PRESS and MEGA-PRESS spectra per cognitive group

Excluded spectra	HC (n=22)	MS (n=60)	CP (n=40)	CI (n=20)
Hippocampus PRESS	0	7	5	2
Thalamus PRESS	1	6	3	3
Hippocampus MEGA-PRESS	4	17	12	5
Thalamus MEGA-PRESS	5	15	10	5

Supplementary table 2 | MRS metabolite concentrations across cognitive groups. Values are mean (SD) or median [IQR] and tests are Welch's ANOVAs. Sample size indicated by 'Glu' also pertains to total Cr and total NAA.

**Right hippocampus**	**HC** (Glu n=22, GABA n=18)	**CP** (Glu n=35, GABA n=28)	**CI** (Glu n=18, GABA n=15)	**P-value**
PRESS SNR	15.2 (4.4)	15.3 (4.2)	11.9 (2.9)	**0.011**
PRESS FWHM	0.096 (0.03)	0.090 (0.03)	0.091 (0.02)	0.73
GABA fit error	11.0 (3.9)	11.4 (4.2)	13.5 (4.5)	0.20
Total Cr (mM)	6.81 (1.35)	7.16 (1.13)	7.59 (0.92)	0.11
Total NAA (mM)	7.91 (1.38)	7.59 (1.29)	7.40 (1.29)	0.46
Glu (mM)	6.72 (1.73)	7.05 (1.41)	7.14 (1.72)	0.66
$GABA^+$ (i.u.)	1.93 (0.60)	2.11 (0.59)	1.73 (0.79)	0.19

**Thalamus**	**HC** (Glu n=21, GABA n=17)	**CP** (Glu n=37, GABA n=30)	**CI** (Glu n=17, GABA n=15)	**P-value**
SNR	18.1 (5.4)	16.9 (4.5)	13.9 (4.4)	**0.023**
FWHM	0.069 (0.033)	0.061 (0.02)	0.069 (0.02)	0.34
GABA fit error	10.5 (3.5)	10.0 (3.7)	11.3 (2.8)	0.53
Total Cr (mM)	7.68 (1.11)	7.54 (1.13)	6.84 (1.62)	0.09
Total NAA (mM)	10.50 (2.00)	9.88 (1.87)	9.48 (2.27)	0.29
Glu (mM)	8.65 (2.42)	8.42 (1.86)	7.55 (2.13)	0.24
$GABA^+$ (i.u.)	2.65 [2.17-3.24]	2.69 [2.06-3.38]	2.68 [1.81-3.29]	0.58

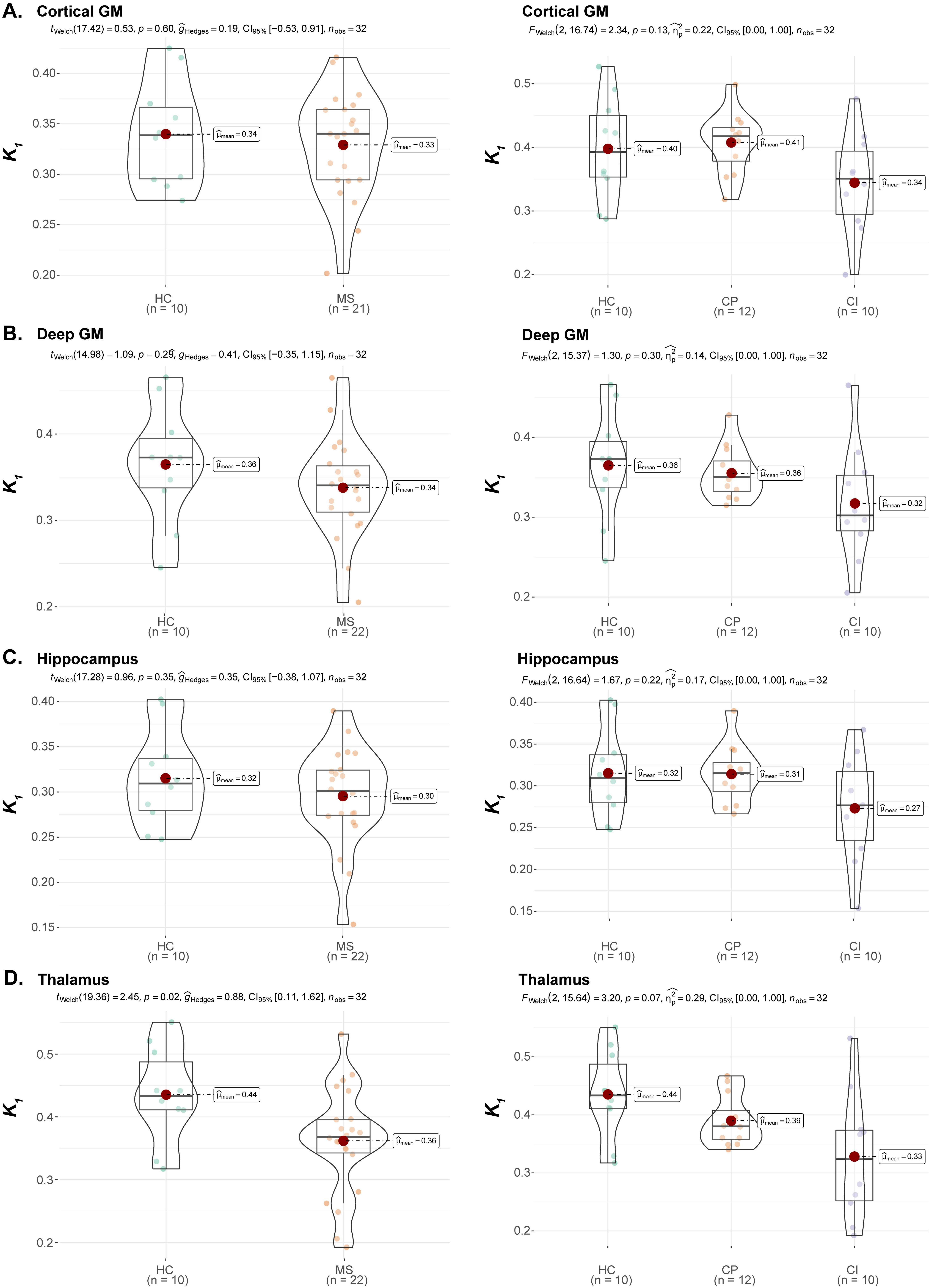

A. Cortical GM
t_Welch(17.42) = 0.53, p = 0.60, ĝ_Hedges = 0.19, CI95% [−0.53, 0.91], n_obs = 32
μ̂_mean = 0.34
μ̂_mean = 0.33
HC (n = 10)
MS (n = 21)
Cortical GM
F_Welch(2, 16.74) = 2.34, p = 0.13, η̂²_p = 0.22, CI95% [0.00, 1.00], n_obs = 32
μ̂_mean = 0.40
μ̂_mean = 0.41
μ̂_mean = 0.34
HC (n = 10)
CP (n = 12)
CI (n = 10)
B. Deep GM
t_Welch(14.98) = 1.09, p = 0.29, ĝ_Hedges = 0.41, CI95% [−0.35, 1.15], n_obs = 32
μ̂_mean = 0.36
μ̂_mean = 0.34
HC (n = 10)
MS (n = 22)
Deep GM
F_Welch(2, 15.37) = 1.30, p = 0.30, η̂²_p = 0.14, CI95% [0.00, 1.00], n_obs = 32
μ̂_mean = 0.36
μ̂_mean = 0.36
μ̂_mean = 0.32
HC (n = 10)
CP (n = 12)
CI (n = 10)
C. Hippocampus
t_Welch(17.28) = 0.96, p = 0.35, ĝ_Hedges = 0.35, CI95% [−0.38, 1.07], n_obs = 32
μ̂_mean = 0.32
μ̂_mean = 0.30
HC (n = 10)
MS (n = 22)
Hippocampus
F_Welch(2, 16.64) = 1.67, p = 0.22, η̂²_p = 0.17, CI95% [0.00, 1.00], n_obs = 32
μ̂_mean = 0.32
μ̂_mean = 0.31
μ̂_mean = 0.27
HC (n = 10)
CP (n = 12)
CI (n = 10)
D. Thalamus
t_Welch(19.36) = 2.45, p = 0.02, ĝ_Hedges = 0.88, CI95% [0.11, 1.62], n_obs = 32
μ̂_mean = 0.44
μ̂_mean = 0.36
HC (n = 10)
MS (n = 22)
Thalamus
F_Welch(2, 15.64) = 3.20, p = 0.07, η̂²_p = 0.29, CI95% [0.00, 1.00], n_obs = 32
μ̂_mean = 0.44
μ̂_mean = 0.39
μ̂_mean = 0.33
HC (n = 10)
CP (n = 12)
CI (n = 10)
K1

Supplementary figure 2 | PVE-corrected influx rate constant (K_1) differences between HC and MS groups. Figures showing the cortical GM **(A)**, deep GM **(B)**, hippocampus **(C)** and thalamus **(D)**. In the thalamus a difference is observed, with PwMS showing lower influx rate constant values. Statistical tests used are Welch's t-test in case of MS-HC comparison (t_{welch}) or Welch's ANOVA (F_{welch}) in case of HC-CP-CI comparison.

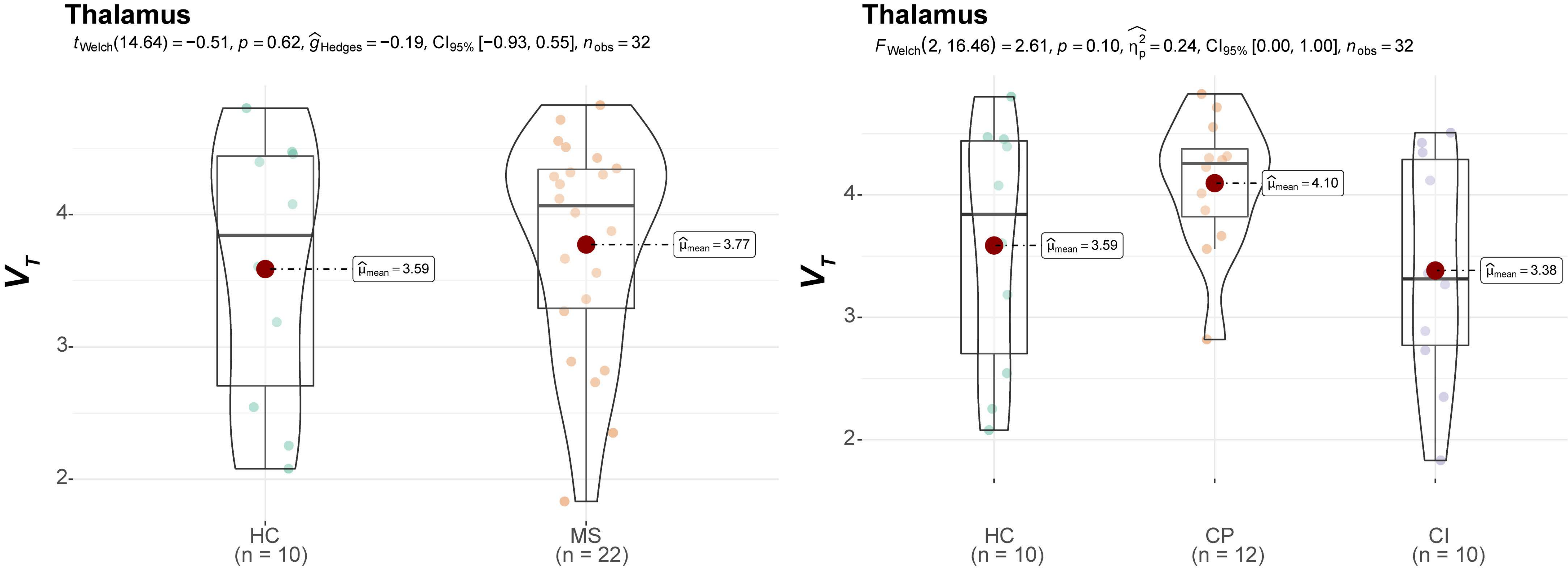

Thalamus
$t_{Welch}(14.64) = -0.51$, $p = 0.62$, $\widehat{g}_{Hedges} = -0.19$, $CI_{95\%}$ [-0.93, 0.55], $n_{obs} = 32$
V_T
4
3
2
$\widehat{\mu}_{mean} = 3.59$
$\widehat{\mu}_{mean} = 3.77$
HC
(n = 10)
MS
(n = 22)
Thalamus
$F_{Welch}(2, 16.46) = 2.61$, $p = 0.10$, $\widehat{\eta_p^2} = 0.24$, $CI_{95\%}$ [0.00, 1.00], $n_{obs} = 32$
V_T
4
3
2
$\widehat{\mu}_{mean} = 3.59$
$\widehat{\mu}_{mean} = 4.10$
$\widehat{\mu}_{mean} = 3.38$
HC
(n = 10)
CP
(n = 12)
CI
(n = 10)

Supplementary figure 3 | PVE-corrected volume of distribution (V_T) values for the thalamus in HC and MS groups. Statistical tests used are Welch's t-test in case of MS-HC comparison (t_{welch}) or Welch's ANOVA (F_{welch}) in case of HC-CP-CI comparison.

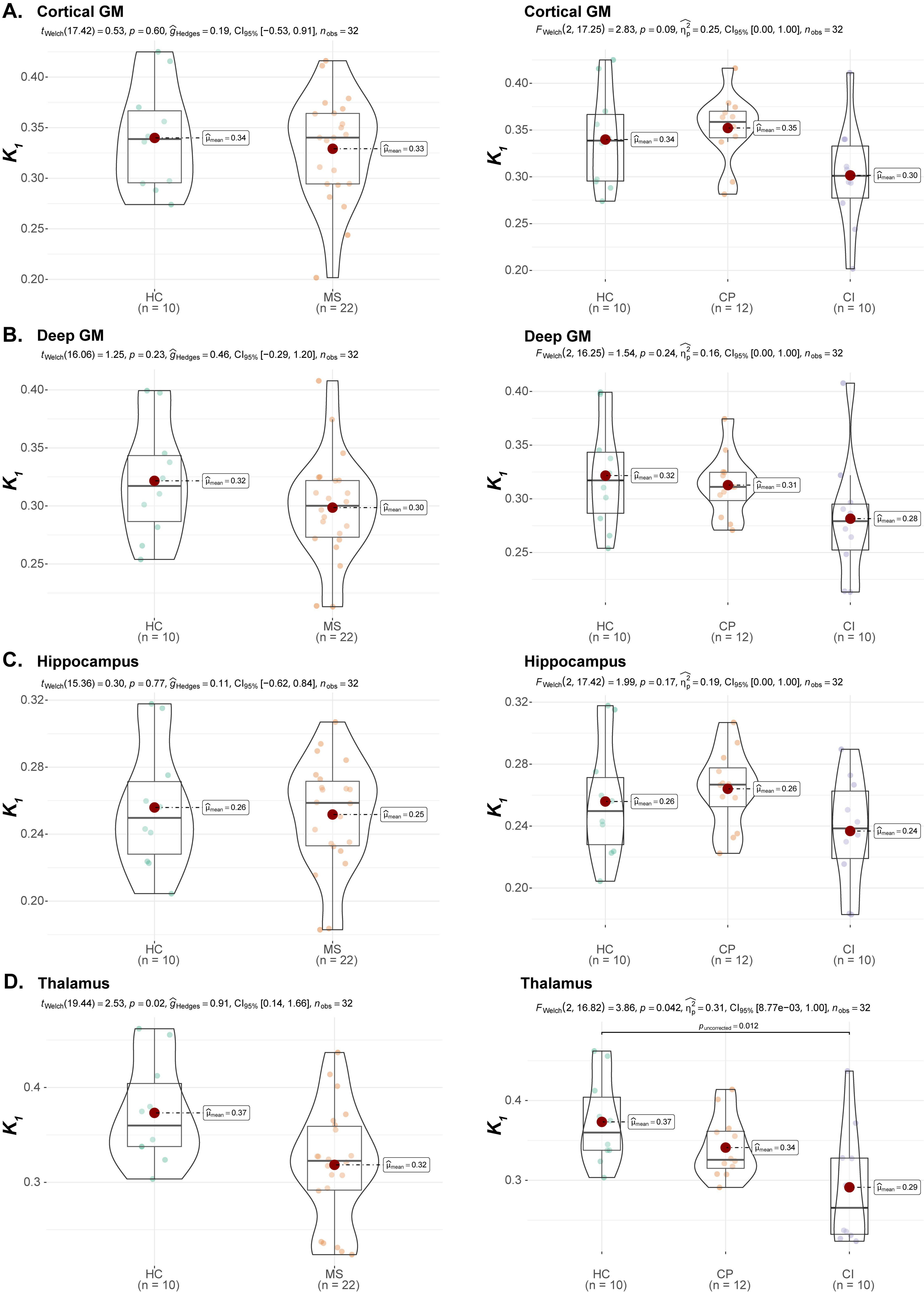
A.
Cortical GM
t_Welch(17.42) = 0.53, p = 0.60, ĝ_Hedges = 0.19, CI95% [−0.53, 0.91], n_obs = 32
μ̂_mean = 0.34
μ̂_mean = 0.33
Cortical GM
F_Welch(2, 17.25) = 2.83, p = 0.09, η̂²_p = 0.25, CI95% [0.00, 1.00], n_obs = 32
μ̂_mean = 0.34
μ̂_mean = 0.35
μ̂_mean = 0.30
B.
Deep GM
t_Welch(16.06) = 1.25, p = 0.23, ĝ_Hedges = 0.46, CI95% [−0.29, 1.20], n_obs = 32
μ̂_mean = 0.32
μ̂_mean = 0.30
Deep GM
F_Welch(2, 16.25) = 1.54, p = 0.24, η̂²_p = 0.16, CI95% [0.00, 1.00], n_obs = 32
μ̂_mean = 0.32
μ̂_mean = 0.31
μ̂_mean = 0.28
C.
Hippocampus
t_Welch(15.36) = 0.30, p = 0.77, ĝ_Hedges = 0.11, CI95% [−0.62, 0.84], n_obs = 32
μ̂_mean = 0.26
μ̂_mean = 0.25
Hippocampus
F_Welch(2, 17.42) = 1.99, p = 0.17, η̂²_p = 0.19, CI95% [0.00, 1.00], n_obs = 32
μ̂_mean = 0.26
μ̂_mean = 0.26
μ̂_mean = 0.24
D.
Thalamus
t_Welch(19.44) = 2.53, p = 0.02, ĝ_Hedges = 0.91, CI95% [0.14, 1.66], n_obs = 32
μ̂_mean = 0.37
μ̂_mean = 0.32
Thalamus
F_Welch(2, 16.82) = 3.86, p = 0.042, η̂²_p = 0.31, CI95% [8.77e−03, 1.00], n_obs = 32
p_uncorrected = 0.012
μ̂_mean = 0.37
μ̂_mean = 0.34
μ̂_mean = 0.29
K1
HC (n = 10)
MS (n = 22)
CP (n = 12)
CI (n = 10)

Supplementary figure 4 | Influx rate constant data (K_1) <u>without</u> PVE-correction. Figures showing the cortical GM **(A)**, deep GM **(B)**, hippocampus **(C)** and thalamus **(D)**. Data are consistent with the PVE-corrected results, confirming lower K_1 in PwMS vs. HC, although these results also indicate lower K_1 in the thalamus of CI PwMS vs. HC. Statistical tests used are Welch's t-test in case of MS-HC comparison (t_{welch}) or Welch's ANOVA (F_{welch}) in case of HC-CP-CI comparison.

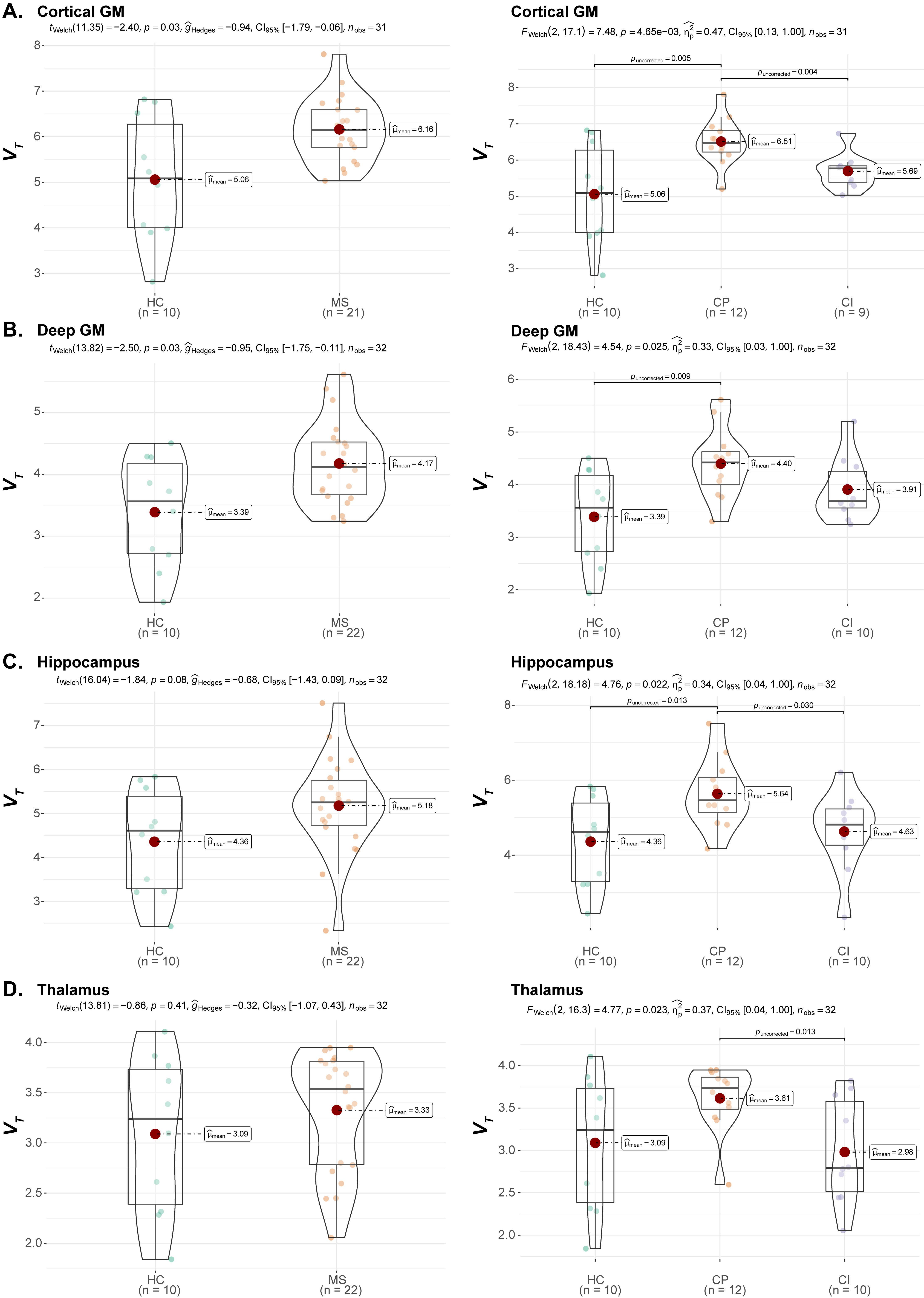

A. Cortical GM
$t_{Welch}(11.35) = -2.40$, $p = 0.03$, $\widehat{g}_{Hedges} = -0.94$, $CI_{95\%}$ [-1.79, -0.06], $n_{obs} = 31$
$\widehat{\mu}_{mean} = 5.06$
$\widehat{\mu}_{mean} = 6.16$
HC (n = 10)
MS (n = 21)
Cortical GM
$F_{Welch}(2, 17.1) = 7.48$, $p = 4.65e-03$, $\widehat{\eta_p^2} = 0.47$, $CI_{95\%}$ [0.13, 1.00], $n_{obs} = 31$
$p_{uncorrected} = 0.005$
$p_{uncorrected} = 0.004$
$\widehat{\mu}_{mean} = 5.06$
$\widehat{\mu}_{mean} = 6.51$
$\widehat{\mu}_{mean} = 5.69$
HC (n = 10)
CP (n = 12)
CI (n = 9)
B. Deep GM
$t_{Welch}(13.82) = -2.50$, $p = 0.03$, $\widehat{g}_{Hedges} = -0.95$, $CI_{95\%}$ [-1.75, -0.11], $n_{obs} = 32$
$\widehat{\mu}_{mean} = 3.39$
$\widehat{\mu}_{mean} = 4.17$
HC (n = 10)
MS (n = 22)
Deep GM
$F_{Welch}(2, 18.43) = 4.54$, $p = 0.025$, $\widehat{\eta_p^2} = 0.33$, $CI_{95\%}$ [0.03, 1.00], $n_{obs} = 32$
$p_{uncorrected} = 0.009$
$\widehat{\mu}_{mean} = 3.39$
$\widehat{\mu}_{mean} = 4.40$
$\widehat{\mu}_{mean} = 3.91$
HC (n = 10)
CP (n = 12)
CI (n = 10)
C. Hippocampus
$t_{Welch}(16.04) = -1.84$, $p = 0.08$, $\widehat{g}_{Hedges} = -0.68$, $CI_{95\%}$ [-1.43, 0.09], $n_{obs} = 32$
$\widehat{\mu}_{mean} = 4.36$
$\widehat{\mu}_{mean} = 5.18$
HC (n = 10)
MS (n = 22)
Hippocampus
$F_{Welch}(2, 18.18) = 4.76$, $p = 0.022$, $\widehat{\eta_p^2} = 0.34$, $CI_{95\%}$ [0.04, 1.00], $n_{obs} = 32$
$p_{uncorrected} = 0.013$
$p_{uncorrected} = 0.030$
$\widehat{\mu}_{mean} = 4.36$
$\widehat{\mu}_{mean} = 5.64$
$\widehat{\mu}_{mean} = 4.63$
HC (n = 10)
CP (n = 12)
CI (n = 10)
D. Thalamus
$t_{Welch}(13.81) = -0.86$, $p = 0.41$, $\widehat{g}_{Hedges} = -0.32$, $CI_{95\%}$ [-1.07, 0.43], $n_{obs} = 32$
$\widehat{\mu}_{mean} = 3.09$
$\widehat{\mu}_{mean} = 3.33$
HC (n = 10)
MS (n = 22)
Thalamus
$F_{Welch}(2, 16.3) = 4.77$, $p = 0.023$, $\widehat{\eta_p^2} = 0.37$, $CI_{95\%}$ [0.04, 1.00], $n_{obs} = 32$
$p_{uncorrected} = 0.013$
$\widehat{\mu}_{mean} = 3.09$
$\widehat{\mu}_{mean} = 3.61$
$\widehat{\mu}_{mean} = 2.98$
HC (n = 10)
CP (n = 12)
CI (n = 10)
V_T

Supplementary figure 5 | Volume of distribution (*V_T*) data <u>without</u> PVE-correction. Figures showing the cortical GM **(A)**, deep GM **(B)**, hippocampus **(C)** and thalamus **(D)**. Data are consistent with the PVE-corrected results, confirming higher V_T in PwMS vs. HC, in particular in CP PwMS. Statistical tests used are Welch's t-test in case of MS-HC comparison (t_{welch}) or Welch's ANOVA (F_{welch}) in case of HC-CP-CI comparison.

Supplemental references

1. Golla SSV, Adriaanse SM, Yaqub M, et al. Model selection criteria for dynamic brain PET studies. *EJNMMI Phys*. 2017;4(1):30. doi:10.1186/s40658-017-0197-0
